# Data on air pollutants and greenery in the city of Yerevan, Armenia

**DOI:** 10.1016/j.dib.2019.104028

**Published:** 2019-05-23

**Authors:** Andranik S. Akopov, Levon A. Beklaryan, Gayane L. Beklaryan

**Affiliations:** a Plekhanov Russian University of Economics, Stremyanny lane, 36, 117997, Moscow, Russian Federation; b Central Economics and Mathematics Institute of Russian Academy of Science, Nachimovski Prosp., 47, 117418, Moscow, Russian Federation

## Abstract

This article contains data related to the research article entitled ‘Agent-based modelling of interactions between air pollutants and greenery using a case study of Yerevan, Armenia’ [1]. These data include the total air pollution and its splitting between different air pollutants in the city of Yerevan, as well as data on agent-vehicles (car clusters) and absorption characteristics of agent-trees. Data and the model that is implemented in the AnyLogic simulation tool are available online at: http://www.runmycode.org/companion/view/3420.

**Table:** Specifications Table

Subject area	Environmental Science
More specific subject area	Ecological Modelling
Type of data	Tables, JPEG files
How data was acquired	Samples were collected and treated at the Central Analytic Laboratory of the Center of Ecological Noosphere Studies of the National Academy of Sciences of Armenia – CENS (cens.am), accredited by ISO/IEC 17025. The collected samples were treated and analysed for air pollutant contents (dust, CO, NOx, SO2, heavy metals, VOCs) through the atomic absorption method (AAnalyst 800, Perkin Elmer, US) (ISO 8573 and ISO 12500, air quality)
Data format	Filtered
Experimental factors	Organic dust, inorganic dust, heavy metals, carbon monoxide (CO), sulphur dioxide (SO2), nitrogen oxides (NOx), carbonates (without volatile organic compounds), volatile organic compounds (VOCs) and other emissions
Experimental features	Samples were collected in the area of the monitoring station, as well as near kindergartens
Data source location	The city of Yerevan. The Republic of Armenia
Data accessibility	The data are attached to this article
Related research article	A.S. Akopov, L.A. Beklaryan, A.K. Saghatelyan, Agent-based modelling of interactions between air pollutants and greenery using a case study of Yerevan, Armenia, Environ. Modell. Softw. 116 (2019) 7–25 [1].

**Table:** 

**Value of the data** • The data are used in the developed agent-based model of interactions between air pollutants and greenery [1] to find the optimal locations of planting various types of trees around kindergartens and emission sources. • The data can be useful for developing different environmental models such as the model of ecological modernization of agent-enterprises [2] and for the validation of genetic optimisation algorithms aggregated with such ecological models [3]. • These data can be used for developing other models of the air pollutants dynamics produced by agent-enterprises and agent-vehicles. • The data show the connection between the number of agent-emissions, their initial radiuses and total air pollution that can be used for developing agent-based environmental models. • The additional value of these data is the possibility of applying in further air pollution researches and for the air quality monitoring in the city of Yerevan, Armenia.

## 1 Data

This dataset contains information on volumes of harmful emissions produced by agent-enterprises and agent-vehicles (car clusters) located in the city of Yerevan, Armenia. In addition, average daily concentrations of some air pollutants observed in 2017 are presented using the map of Yerevan.

The basic dataset contains main characteristics of agent-enterprises of the city of Yerevan, Armenia is presented in Table 1. These data are used for developing the agent-based model of interactions between air pollutants and greenery in the city of Yerevan, Armenia [1] to simulate the behaviour of agent-enterprises. In particular, the number of agent-emissions and their initial radiuses are the main characteristics of agent-enterprises in the model. These values affect the predictive daily air pollution concentration in the city. Table 2 presents the detailed data on different air pollutants produced by agent-enterprises in the city of Yerevan, Armenia. As shown in the work [1], different agent-emissions contain various types of air-pollutants (e.g. the inorganic dust, carbon monoxide, nitrogen oxides (NOx), sulphur dioxide (SO2), etc.). The basic dataset contains main characteristics of agent-vehicles of the city of Yerevan, Armenia is presented in Table 3. These data are used for developing the agent-based model of interactions between air pollutants and greenery in the city of Yerevan, Armenia to simulate the behaviour of agent-vehicles. In particular, the total number of cars and agent-vehicles (car clusters) distributed by urban areas allows to simulate the heterogeneous concentration of harmful emissions produced by vehicles in the city. This is extremely important for kindergartens and other protected urban areas (e.g. schools, hospitals) located near the roads. Table 4 presents the maximum values of the air pollution absorption produced by different types of trees in Yerevan. These data are needed to simulate the dynamics of absorption processes in the model of the interaction between air pollutants and greenery and to find optimal locations of planting greenery in the city [1], [3]. Table 5 presents data on green spaces in Yerevan (ha.), the areas of which were estimated in June 2017.

**Table 1:** Basic dataset of agent-enterprises of the city of Yerevan, Armenia.

No.	Names of agent-enterprises	Number of agent-emissions produced for one event	Averaged initial radius of agent-emissions, metres	Total air pollution (2007–2014), tonnes
1	ARMROSGAZPROM YEREVAN GGM	20	100	52201.9
2	ARMROSGAZPROM	20	61	11752.2
3	YEREVAN WATERPROOF ELECTRONIC EQUIPMENT	20	53	3482.6
4	ARMENIAN MOLYBDENUM PRODUCTION	20	53	2984.9
5	PURE IRON PLANT	20	53	2942.6
6	V-STAR	20	53	2862.0
7	AVTOGAS	10	51	850.7
8	VARDASHEN MINING	8	50	401.7
9	AMELIA MINING COMPANY	8	50	400.0
10	GRAND CANDY	8	50	393.7
11	YEREVAN M. AFTER HERATSI JET! MEDICAL CENTER: UNIVERSAL	8	50	362.0
12	PLASTERER	8	50	337.7
13	ALABASTER	8	50	305.9
14	YEREVAN POULTRY	8	50	301.5
15	MIKA CORPORATION	8	50	256.8
16	NAIRIT PLANT	8	50	256.2
17	RUSAL ARMENAL	8	50	235.2
18	ROBEL	8	50	227.2
19	GRIAR	8	50	206.0
20	AVAN SALT PLANT	6	50	193.5
21	CANVAS	6	50	188.7
22	CRUSHED MUSA	6	50	188.3
23	EUROPE	6	50	186.4
24	ARMENIA-INTERNATIONAL AIRPORTS	6	50	157.9
25	ALEX-GRIG	6	50	157.0
26	ARMENIAN TITAN PRODUCTION	6	50	149.5
27	ELECTRIC NETWORKS OF ARMENIA	6	50	147.0
28	MLL INDUSTRIES	6	50	139.7
29	BEER OF YEREVAN	6	50	136.8
30	YEREVAN STATE UNIVERSITY	6	50	114.6
31	VAHAGN AND SAMVEL	6	50	107.7
32	HACHAR	6	50	107.7
33	SH INFORM	6	50	101.2
34	SOFTTECH	4	50	99.2
35	ELSE STYLE	4	50	95.3
36	ANAR	4	50	89.2
37	PROSHYAN BRANDY FACTORY	4	50	86.6
38	ARPA-SEVAN	4	50	85.4
39	YEREVANI ARARAT COGNAC ENTERPRISE	4	50	78.0
40	STONMAN	4	50	72.1
41	BASALT-G. A. R.	4	50	69.9
42	GIDROIZOL	4	50	66.8
43	FLASH	4	50	61.0
44	COCA-COLA HBK ARMENIA	4	50	59.8
45	VT TRADE	4	50	57.8
46	METAL STRUCTURES PLANT	4	50	47.3
47	QUARTZ	4	50	44.7
48	ARABKIR CHEMICAL CLEANING	4	50	43.5
49	EVROTERM	4	50	40.9
50	LIMITED BASALT	4	50	37.0
51	STONE AND SAND ARE NOT MINERALS	4	50	34.9
52	SHEN HOLDING	4	50	32.5
53	ROOK AND RUBEN	4	50	30.8
54	ROAD Builder	4	50	30.2
55	IVECO GROUP	4	50	28.9
56	CONCRETE	4	50	26.3
57	SHARUR	4	50	25.9
58	BABYC-90	4	50	24.3
59	GRAND TOBACCO	4	50	23.9
60	ST SERVICE	4	50	23.6
61	DAUGHTER MARIANNA	4	50	23.5
62	ECOPROTECT	4	50	22.8
63	DOST INTERNATIONAL	4	50	22.8
64	KARTON-TARA	4	50	22.3
65	A & G	4	50	21.6
66	GIKAR	4	50	20.0
67	EREBUNI MEDICAL CENTER	4	50	18.7
68	ARPA	4	50	18.6
69	A. HE. OPERA AND BALLET THEATRE	4	50	17.4
70	ARMENIAN TITANIUM PRODUCTION	4	50	12.9
71	DELTA FRAGMENTS	4	50	12.5
72	HOVNANIAN INTERNATIONAL	4	50	11.1
73	AVA-2000	4	50	10.8
74	ASPHALT	4	50	10.7
75	SHIN-MEN'S	4	50	10.6
76	ARKADY-VLADIMIR	4	50	10.2
77	SPARKLING WINES PLANT	4	50	10.2
78	ARMCABLE	4	50	9.7
79	TAMARA	4	50	9.6
80	DIA	4	50	9.5
81	DALMA INVEST	4	50	9.5
82	EREBUNI WINE-BRANDY, MR.	4	50	9.3
83	SEUA	4	50	9.1

**Table 2:** The dataset on different air pollutants by agent-enterprises of the city of Yerevan, Armenia.

Names of agent-enterprises	Total air pollution (2007–2014), tonnes	Inorganic dust, tonnes	Carbon monoxide, tonnes	Nitrogen oxides (NOx) tonnes	Organic dust, tonnes	Total heavy metals, tonnes	Sulphur dioxide (SO2), tonnes	Carbonates (without volatile organic compounds) tonnes	Volatile organic compounds (VOC), tonnes	Other, tonnes
ARMROSGAZPROM YEREVAN GGM	52201.86	0.00	18.36	5.96	0.00	0.00	0.00	51935.95	241.59	0.00
ARMROSGAZPROM	11752.15	0.00	15.05	113.30	0.00	0.00	0.00	11601.70	22.10	0.00
YEREVAN DECREE	3482.63	0.00	0.00	3472.22	10.40	0.00	0.00	0.00	0.00	0.01
ARMENIAN MOLYBDENUM PRODUCTION	2984.86	368.10	424.38	146.77	0.00	0.00	2045.61	0.00	0.00	0.00
PURE IRON PLANT	2942.62	25.85	244.40	32.62	0.00	25.94	2606.74	0.00	0.00	7.08
V.-ST.	2861.98	0.00	0.00	0.00	0.00	0.00	0.00	2851.42	10.56	0.00
AVTOGAS	850.71	0.00	0.00	0.00	0.00	0.00	0.00	849.54	1.16	0.00
GIVING MINE	401.74	326.70	0.00	0.00	75.00	0.00	0.00	0.00	0.00	0.04
UPTIME	400.02	400.02	0.00	0.00	0.00	0.00	0.00	0.00	0.00	0.00
GRAND CANDY	393.68	0.00	299.30	91.30	0.00	0.00	0.00	0.00	0.53	2.54
The YEREVAN M. HERATSI THEM. VET. MEDICAL. MOVIE.	362.03	0.00	284.18	77.85	0.00	0.00	0.00	0.00	0.00	0.00
PLASTERER	337.70	106.20	178.60	52.90	0.00	0.00	0.00	0.00	0.00	0.00
ALABASTER	305.88	87.26	163.35	55.27	0.00	0.00	0.00	0.00	0.00	0.00
YEREVAN POULTRY	301.46	0.00	16.31	5.95	3.00	0.00	0.00	92.20	0.00	184.00
MIKA CORPORATION	256.80	0.00	0.00	0.00	0.00	0.00	0.00	256.80	0.00	0.00
THE PLANT	256.20	14.65	3.04	0.86	0.96	0.00	0.00	0.00	184.54	52.16
RUSAL ARMENAL	235.15	53.76	61.81	32.92	28.47	0.00	0.00	0.00	0.00	58.20
REL	227.17	0.00	0.00	0.00	0.00	0.00	0.00	226.91	0.26	0.00
IN THE BUILDING	206.02	173.25	17.86	5.96	4.97	0.00	0.00	0.00	3.82	0.16
AVAN SALT PLANT	193.45	12.30	128.80	51.85	0.46	0.00	0.00	0.00	0.00	0.04
CANVAS	188.69	0.00	134.93	53.76	0.00	0.00	0.00	0.00	0.00	0.00
CRUSHED MUSA	188.33	0.00	0.00	0.00	0.00	0.00	0.00	188.33	0.00	0.00
EUROPE	186.41	3.21	71.51	13.48	79.68	0.00	16.29	0.00	1.23	1.01
ARMENIA-INTERNATIONAL AIRPORTS	157.86	0.00	119.65	38.21	0.00	0.00	0.00	0.00	0.00	0.00
ALEX-GRIG	157.00	0.00	121.26	35.74	0.00	0.00	0.00	0.00	0.00	0.00
ARMENIA TITANS PRODUCTION	149.48	66.30	62.41	20.77	0.00	0.00	0.00	0.00	0.00	0.00
ELECTRIC NETWORKS OF ARMENIA	147.03	39.76	26.22	7.98	0.00	0.00	0.00	0.00	62.04	11.03
MLL INDUSTRIES	139.70	139.70	0.00	0.00	0.00	0.00	0.00	0.00	0.00	0.00
BEER OF YEREVAN	136.79	0.04	61.80	62.53	1.69	0.00	0.00	0.00	1.58	9.15
YEREVAN STATE UNIVERSITY	114.57	0.00	82.76	31.81	0.00	0.00	0.00	0.00	0.00	0.00
VAHAGN AND SAMVEL	107.69	82.27	3.13	3.83	0.00	0.00	0.00	8.24	0.00	10.22
MAKAR	107.67	34.80	58.21	14.66	0.00	0.00	0.00	0.00	0.00	0.00
INFORM	101.23	65.60	30.41	4.72	0.00	0.00	0.00	0.50	0.00	0.00
SOFTWARE	99.20	0.00	74.00	25.20	0.00	0.00	0.00	0.00	0.00	0.00
ELSIE STEELE	95.30	0.00	0.00	0.00	0.00	0.00	0.00	95.18	0.11	0.00
ANAR	89.19	78.30	6.49	2.90	0.00	0.00	0.00	1.50	0.00	0.00
PROSHYAN BRANDY FACTORY	86.63	0.00	46.13	16.11	0.00	0.00	0.00	0.00	24.35	0.04
ARPA-SEVAN	85.39	84.60	0.00	0.00	0.00	0.00	0.00	0.00	0.00	0.79
YEREVAN BRANDY-WINE-VODKA FACTORY	77.99	0.00	57.96	20.03	0.00	0.00	0.00	0.00	0.00	0.00
STONMAN	72.10	72.10	0.00	0.00	0.00	0.00	0.00	0.00	0.00	0.00
BASALT-G. A. R.	69.90	69.90	0.00	0.00	0.00	0.00	0.00	0.00	0.00	0.00
GIDROIZOL	66.79	0.05	11.83	4.04	0.00	0.00	0.00	50.40	0.00	0.47
FLASH	61.00	0.00	0.00	0.00	0.00	0.00	0.00	61.00	0.00	0.00
COCA-COLA HBK ARMENIA	59.76	0.00	45.30	14.46	0.00	0.00	0.00	0.00	0.00	0.00
VT TRADE	57.80	0.00	0.00	0.00	57.80	0.00	0.00	0.00	0.00	0.00
METAL STRUCTURES PLANT	47.31	33.10	4.90	1.93	0.00	0.00	0.00	3.50	0.00	3.88
QUARTZ	44.70	41.00	3.00	0.70	0.00	0.00	0.00	0.00	0.00	0.00
IN BUILDING DRY CLEANING	43.50	0.00	0.35	0.11	0.00	0.00	0.00	0.00	43.03	0.00
EVROTERM	40.87	0.00	29.24	11.63	0.00	0.00	0.00	0.00	0.00	0.00
LIMITED BASALT	37.00	28.65	7.21	1.13	0.00	0.00	0.00	0.00	0.00	0.01
STONE AND SAND ARE NOT MINERALS	34.89	34.89	0.00	0.00	0.00	0.00	0.00	0.00	0.00	0.00
SHEN HOLDING	32.46	17.84	11.47	1.65	0.08	0.00	0.00	0.00	1.42	0.00
ROOK AND RUBEN	30.80	30.80	0.00	0.00	0.00	0.00	0.00	0.00	0.00	0.00
ROAD Builder	30.20	17.17	8.23	2.39	0.00	0.00	0.00	2.42	0.00	0.00
IVECO GROUP	28.90	28.90	0.00	0.00	0.00	0.00	0.00	0.00	0.00	0.00
CONCRETE	26.33	26.33	0.00	0.00	0.00	0.00	0.00	0.00	0.00	0.00
SHARUR	25.92	24.00	1.44	0.48	0.00	0.00	0.00	0.00	0.00	0.00
BOBBY-90	24.29	0.00	18.36	5.93	0.00	0.00	0.00	0.00	0.00	0.00
GRAND TOBACCO	23.92	0.00	16.52	6.14	1.26	0.00	0.00	0.00	0.00	0.00
ES TI SERVICE	23.60	23.60	0.00	0.00	0.00	0.00	0.00	0.00	0.00	0.00
DAUGHTER MARIANNA	23.46	0.00	17.80	5.66	0.00	0.00	0.00	0.00	0.00	0.00
ECOPROTECT	22.77	0.90	13.94	6.23	0.80	0.00	0.00	0.00	0.90	0.00
DOST INTERNATIONAL	22.75	0.00	16.34	6.41	0.00	0.00	0.00	0.00	0.00	0.00
KARTON-TARA	22.26	0.00	14.73	7.53	0.00	0.00	0.00	0.00	0.00	0.00
A & G	21.61	0.00	15.67	5.34	0.60	0.00	0.00	0.00	0.00	0.00
VIKAR	20.00	20.00	0.00	0.00	0.00	0.00	0.00	0.00	0.00	0.00
EREBUNI MEDICAL CENTER	18.74	0.00	13.47	5.27	0.00	0.00	0.00	0.00	0.00	0.00
ARPA	18.61	0.11	2.60	1.00	0.00	0.00	0.00	0.13	14.74	0.02
A. HE. OPERA AND BALLET THEATRE	17.36	0.00	1.06	16.30	0.00	0.00	0.00	0.00	0.00	0.00
MAVA IMPULSE	12.88	0.00	10.80	1.88	0.20	0.00	0.00	0.00	0.00	0.00
DELTA FRAGMENTS	12.49	12.49	0.00	0.00	0.00	0.00	0.00	0.00	0.00	0.00
HOVNANIAN INTERNATIONAL	11.06	6.41	3.47	1.19	0.00	0.00	0.00	0.00	0.00	0.00
AVA-2000	10.77	4.03	5.16	0.86	0.00	0.00	0.00	0.72	0.00	0.00
ASPHALT	10.74	2.49	4.36	0.79	0.00	0.00	0.00	3.10	0.00	0.00
SHIN-MEN'S	10.64	0.00	7.93	2.71	0.00	0.00	0.00	0.00	0.00	0.00
ARKADY-VLADIMIR	10.19	6.50	2.74	0.95	0.00	0.00	0.00	0.00	0.00	0.00
SPARKLING WINES PLANT	10.16	0.00	7.49	2.67	0.00	0.00	0.00	0.00	0.00	0.00
Was ICAL	9.65	0.00	1.57	0.63	1.55	0.00	0.00	0.00	5.71	0.20
TAMARA	9.56	9.21	0.11	0.20	0.00	0.00	0.00	0.05	0.00	0.00
DIA	9.54	3.92	4.26	1.36	0.00	0.00	0.00	0.00	0.00	0.00
DALMA INVEST	9.51	0.00	7.20	2.31	0.00	0.00	0.00	0.00	0.00	0.00
EREBUNI WINE-BRANDY, MR.	9.30	0.00	6.00	2.10	1.20	0.00	0.00	0.00	0.00	0.00
SEUA	9.13	0.00	7.00	2.11	0.02	0.00	0.00	0.00	0.00	0.00

**Table 3:** Basic dataset of agent-vehicles of the city of Yerevan, Armenia.

No.	Administrative name of urban area	Total number of cars	Number of agent-vehicles
1	ACHAPNYAK	30300	101
2	ARABKIR	36300	121
3	AVAN	12000	40
4	DAVTASHEN	12300	41
5	EREBUNI	30300	101
6	KANAKER-ZEYTUN	24600	82
7	CENTER	43200	144
8	MALATIYA-SEBASTIYA	38100	127
9	NORK-MARASH	3600	12
10	NOR-NORK	31800	106
11	NUBARASHEN	2100	7
12	SHENGAVIT	35400	118
	**TOTAL**	**300000**	**1000**

**Table 4:** Estimated maximum air pollution absorption per tree in Yerevan (kg/year).

Types of air pollutants	POPLAR	OAK	MAPLE	PINE	ULMUS
Carbon dioxide (CO2)	22	21	20	20	21
Organic dust, kilo	15	15	15	15	90
Inorganic dust, kilo	15	15	15	15	90
Total heavy metals, kilo	0.5	0.5	0.5	0.5	0.5
Sulphur dioxide (SO2), kilo	2	2	2	2	2
Carbon monoxide, kilo	1	1	1	1	1
Nitrogen oxides (NOx), kilo	3.5	3.5	3.5	3.5	3.5
Carbonates (without volatile organic compounds), kilo	1	1	1	1	1
Volatile organic compounds (VOC), kilo	0.9	0.9	0.9	3	0.9

**Table 5:** Green spaces in Yerevan (ha.).

No.	Administrative name of urban area	Area (ha.)
1	ACHAPNYAK	441
2	ARABKIR	575
3	AVAN	358
4	CENTER	447
5	DAVTASHEN	174
6	EREBUNI	1120
7	KANAKER-ZEYTUN	315
8	MALATIYA-SEBASTIYA	758
9	NORK-MARASH	264
10	NOR-NORK	499
11	NUBARASHEN	465
12	SHENGAVIT	1535
	**Total urban area with greenery**	**6952**

**Fig. 1:**
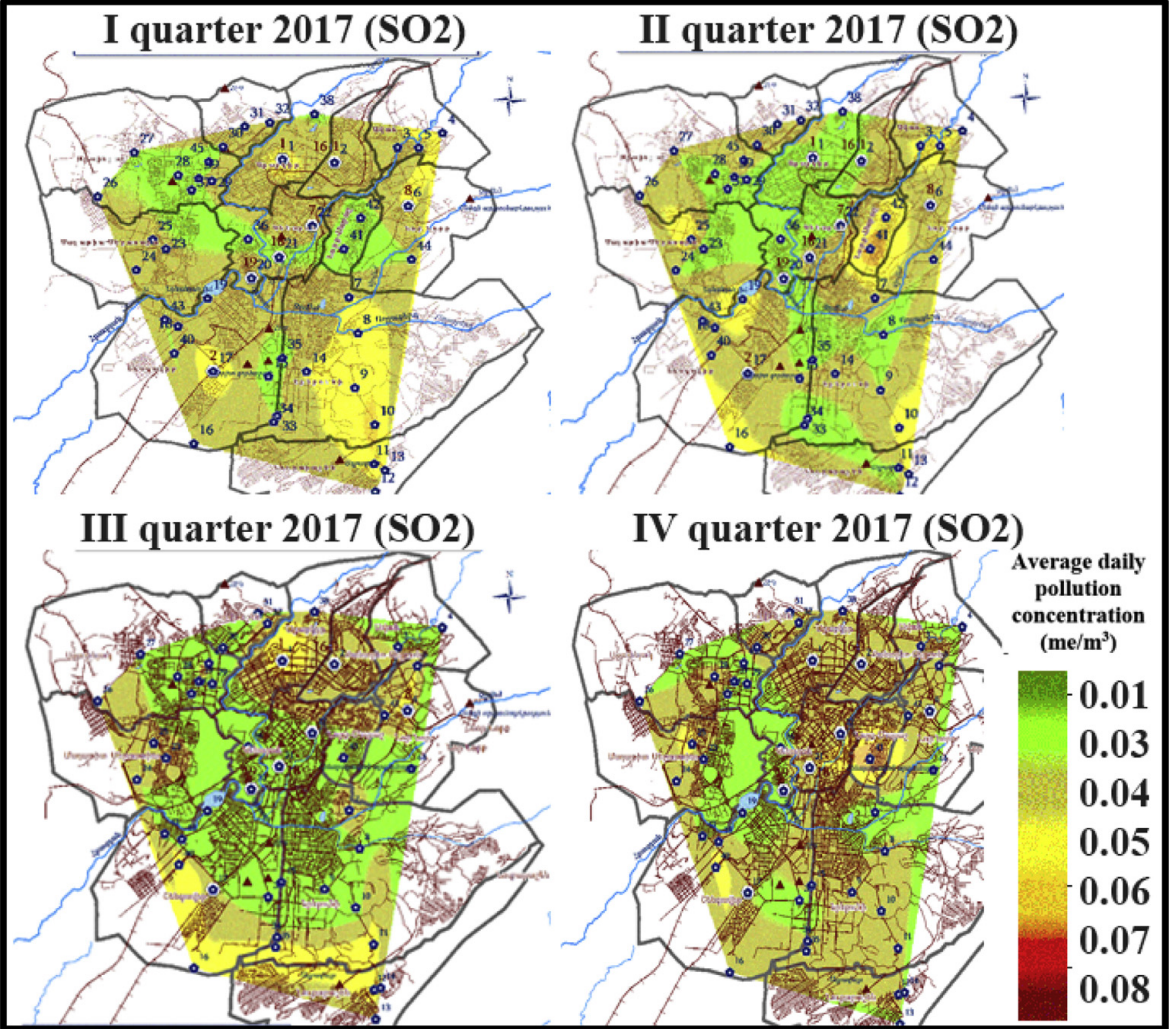
The example of samples on the actual observation of the average daily air pollutant concentrations of SO2 in the city of Yerevan, Armenia in 2017.

**Fig. 2:**
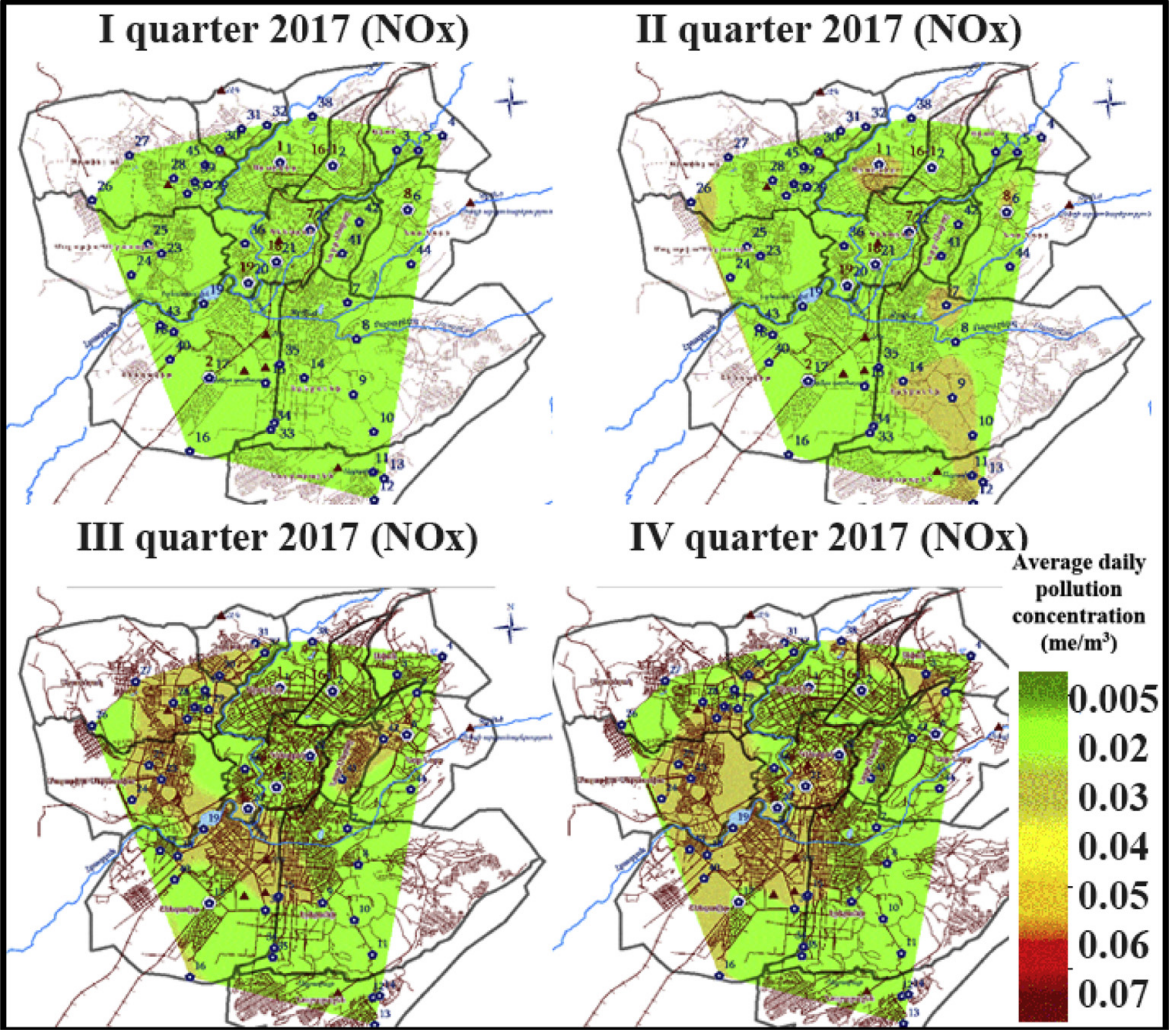
The example of samples on the actual observation of the average daily air pollutant concentrations of NOx in the city of Yerevan, Armenia in 2017.

The actual observation of the average daily air pollutant concentrations of SO2, NOx in the city of Yerevan, Armenia are represented in Fig. 1, Fig. 2. These data provided by the Armenian center of monitoring and information environment (www.armmonitoring.am) and are used for the model validation [1]. In addition, such observations can be useful to estimate the air quality in different urban areas.

## 2 Experimental design, materials, and methods

### 2.1 Method description

Samples were collected and treated at the Central Analytic Laboratory of CENS, accredited by ISO/IEC 17025. The laboratory has modern equipment, including a Serinus 30 carbon monoxide analyser, Serinus 40 analyser, Serinus 40 nitrogen oxides analyser, Serinus 51 sulphur dioxide analyser, VOC72 M Gas Chromatography Volatile Organic Compounds (BTEX) analyser, and dust analyser OPASTOP GP4000HD. The collected samples were treated and analysed for air pollutant contents (dust, CO, NOx, SO2, heavy metals, VOCs) through the atomic absorption method (AAnalyst 800, Perkin Elmer, US) (ISO 8573 and ISO 12500, air quality).

### 2.2 Observations description

The regular monitoring of the dust, sulphur dioxide, nitrogen oxides, carbon monoxide and other typos of pollutants is conducted in the city of Yerevan, Armenia. At the same time, both automatic observations and sampling in different urban areas with subsequent laboratory studies are carried out. The examples of the actual observation of the average daily air pollutant concentrations of SO2, NOx in the city of Yerevan, Armenia are represented in Fig. 1, Fig. 2.
